# “I don't grieve as much as I used to”: A qualitative study on parents of children with rare and undiagnosed conditions navigating grief in the context of uncertainty

**DOI:** 10.1002/jgc4.70149

**Published:** 2025-11-29

**Authors:** Tara Maria Hoffmann, Bettina Friedrich, Celine Lewis

**Affiliations:** ^1^ Population, Policy and Practice Department UCL Great Ormond Street Institute of Child Health London UK; ^2^ Department of Paediatrics Medical Center Hamburg‐Eppendorf (UKE) Hamburg Germany; ^3^ Department of Epidemiology and Health Care UCL London UK; ^4^ London North Genomic Laboratory Hub Great Ormond Street Hospital for Children NHS Foundation Trust London UK

**Keywords:** ambiguous loss, chronic sorrow, coping with grief, family grief, genomic sequencing, grief, mental health, parent caregivers, psychosocial support, rare diseases, uncertainty, undiagnosed conditions

## Abstract

The aim of this qualitative interview study was to explore the lived experiences of parents, experiencing high anxiety and poorer quality‐of‐life/family functioning, caring for a child with a rare and undiagnosed condition. Data analysis led to the generation of a substantial corpus of insights focusing on how parents cope with grief amidst the uncertainty surrounding their child's condition. Whereas much is known about grief related to death in pediatric cancer patients, research focusing on grief in the area of rare and undiagnosed conditions is sparse. We conducted semi‐structured interviews with 24 parents of children affected by a rare and undiagnosed condition undergoing whole genome sequencing (WGS) through the Genomic Medicine Service (GMS) in England and Wales. Participants were purposively sampled based on scores to validated psychological measures. We used reflexive thematic analysis, situated within an interpretivist and post‐positivist research paradigm, to explore the data. The central organizing concept was named “Navigating Grief In The Context Of Uncertainty.” This overarching theme describes how these parents grieve the loss of the envisioned future they held while navigating an unpredictable reality shaped by their child's undiagnosed condition. Our findings also highlight the “*potential ongoingness*” of grief, although it may change over time. Parents adapt through constructive reframing, seeking meaning and acceptance, and fostering resilience all of which we found to aid in coping. Understanding the grieving process, particularly the role of uncertainty, is essential for improving the clinical support provided to families affected by rare and undiagnosed conditions and for designing future psychological intervention strategies that address parents who grieve the loss of their anticipated family life.

**What is known about this topic**

Grief in parents of children with rare and undiagnosed conditions is less understood compared to grief in contexts like pediatric oncology, where clarity about prognosis can sometimes support hope, providing a path towards meaning making and resilience. In contrast, these parents face ongoing uncertainty about their child's symptoms and life trajectory, requiring them to perpetually adjust their expectations and navigate a complex, evolving grieving process.

**What this paper adds to the topic**

Our findings highlight the complex and emotional journey, characterized by grief, acceptance, and adaptation for this particular cohort of parents against a background of broader systemic failures to adequately support families and recognize diverse developmental trajectories. Our study expands existing grief frameworks by emphasizing that grief and acceptance are not opposites, but part of a complex and evolving process for families navigating rare and undiagnosed conditions. We also discuss approaches for supporting parents through this process.

## 1 BACKGROUND

### 1.1 The emotional impact of parenting a child with a rare and undiagnosed condition

Parents caring for a child with a rare condition, which refers to a medical condition that affects fewer than 1 in 2000 people (Department of Health and Social Care, 2021) have been found to experience clinically relevant anxiety and depression, influencing quality of life (Boettcher et al., 2021; Sandilands et al., 2022; Williams, 2025). A systematic review of parenting stress among caregivers of children with chronic illness found that caregivers reported significantly greater general parenting stress than caregivers without a child with a chronic illness (*p* ≤ 0.0001) using the Parenting Stress Index as a comparator, and it was associated with poorer psychological adjustment and greater parental responsibility for treatment management (Cousino & Hazen, 2013). A more recent systematic review, focused on parents of children with rare congenital genetic disorders, identified a wide range of emotional experiences, such as shock, anxiety and fear, lack of control, defencelessness, depression or loss, denial, self‐blame and guilt, helplessness, and distress (von der Lippe et al., 2022).

Notably, however, reported lived experiences have also included positive aspects of parenting a child with a rare condition. In a systematic review of the experiences of parenting a child with intellectual disability, the authors identified themes relating to positive change, increased personal strength, growth, and development largely related to parental intrapersonal orientation (Beighton & Wills, 2019). In the systematic review by Von der Lippe et al., alongside some of the more challenging emotional reactions, the authors identified protective factors that had strengthened parents' coping mechanisms (von der Lippe et al., 2022). These included having engaged and understanding healthcare professionals, the development of self‐reliance and trust in their ability to cope with problems, and a process of normality reconstruction, incorporating the child's condition with its consequences, which appeared to give parents a sense of control over their situation (von der Lippe et al., 2022).

Research around the experience of parents caring for children with both rare and undiagnosed conditions, where the underlying cause of the condition is not yet established, is more sparse but has highlighted that parents experience guilt, fear, and frustration at the lack of information about the condition (Skirton et al., 2010). In our quantitative survey study of the psychosocial profiles of parents of children with rare and undiagnosed conditions conducted as part of this programme of research, we found that the mean score on the Generalized Anxiety Disorder [GAD‐7] measure was 5.31 and 22% of parents of children scored as having moderate or severe anxiety. Multivariable analysis indicated that higher anxiety scores were significantly associated with lower resilience and lower tolerance for uncertainty (Patel et al., 2025a). In a similar study of parents of children with undiagnosed conditions aged 1–18 (mean age 7.83 years) conducted in the United States, the mean score on the Patient Health Questionnaire [PHQ‐9] which measures depression was 4.8 and around 34% met the criteria for mild to moderate depression, and the mean score on the GAD‐7 was 4.85, with 41% reaching the criteria for mild, moderate, or severe anxiety (McConkie‐Rosell et al., 2018). In the United States, parents of school‐age children (aged between 5 and 12 years) from the general population had a mean PHQ score of 4.0 and a mean GAD‐7 score of 3.4 (Sequeira et al., 2021). Qualitative research has also revealed that parents of undiagnosed children have to push or fight to access services including educational support, therapeutic services and specialized equipment, in part due to the lack of a “label” which can make administrative tasks more complicated, leaving parents emotionally exhausted (Aldiss et al., 2021; Skirton et al., 2010; Yanes et al., 2017).

### 1.2 Research around grief in the rare disease literature

In the rare disease literature, grief and loss, including loss of perceived normality and loss of hope or anticipated grief related to loss of an envisioned future, have been identified as responses associated with their child's condition (Gurasashvili et al., 2023; Nevin et al., 2022). Research on family grief before a loved one's death in life‐limiting illness highlights inconsistencies in conceptualizing related constructs, with pre‐death grief—encompassing anticipatory grief (future‐focused distress) and illness‐related grief (present‐focused loss)—offering a unified framework for study (Singer et al., 2022). Parents of children with life‐threatening conditions frequently experience what has been described as “anticipatory grief” related to their child's condition and death, which can fluctuate and persist over time reemerging with new symptoms or hurdles associated with the condition (Najafi et al., 2022). Grief as a response to loss may involve phases such as denial, anger, bargaining, depression, acceptance, and meaning‐making, though not everyone experiences all phases, nor do they always follow a specific order (Kessler, 2019). Each individual copes with grief in their own unique way (Kessler, 2019). Coping with grief in the context of uncertainty surrounding rare conditions presents a complex task, for both families and clinicians, of balancing hope with realistic expectations, which can result in recurring disappointments (Bye et al., 2022; Gurasashvili et al., 2023; Nevin et al., 2022; Palmer et al., 2022). Ongoing uncertainty caused by the absence of a clear diagnosis may contribute to a persistent and cyclical grieving process. Parents may also experience what is known as “chronic sorrow” which refers to the enduring, recurrent grief experienced by caregivers of individuals with chronic illnesses or disabilities (Lindgren et al., 1992).

### 1.3 Rationale for this study

The overarching aim of this qualitative study was to explore the mental health impact of parenting a child with a rare and undiagnosed condition, and is embedded within a mixed‐methods longitudinal patient‐centred evaluation of the Genomic Medicine Service (Lewis et al., 2021). Following data collection, data analysis revealed a significant data corpus related to the concept of grief. Although there is substantial knowledge about grief and bereavement in the context of pediatric oncology (Lichtenthal et al., 2015; Pedraza et al., 2023) or when facing the prospect of a child's death, less is understood about the grieving process experienced by parents caring for a child with a rare and undiagnosed condition with an uncertain outcome. For instance, in pediatric cancer studies, it was found that prognostic disclosure, even when regarded as distressing, does not diminish hope and can instead foster it by providing clarity and support to grieving parents (Mack et al., 2006, 2007). The situation may be different for parents caring for a child with a condition of uncertain outcome, who may know that life expectancy may be limited, but who, in the absence of a diagnosis, are unsure how to assess the severity of their child's recurrent symptoms (Aldiss et al., 2021). Other aspects of uncertainty related to rare and undiagnosed conditions include the uncertainty families face in family planning as the diagnosis, and hence the recurrence rate for future children, is unknown (Madeo et al., 2012). For many rare conditions, effective treatments do not exist and there are very few paths to identifying treatment (Yanes et al., 2017). Additionally, the healthcare providers that parents encounter may not be familiar with how to navigate their child's condition and manage their symptoms, as well experience a lack of coordinated care, which can contribute to uncertainty (Aldiss et al., 2021). Finally, due to the rarity of these conditions, families often feel there is a lack of community and support available to them—which is important in managing grief—particularly when the child remains undiagnosed (Aldiss et al., 2021; Gurasashvili et al., 2023).

The emotionally complex process of coping with uncertainty can also extend to parents' hopeful expectations about their anticipated family life trajectory. In their qualitative study of the experience of parenting a child with an undiagnosed condition, Lewis et al. (2010) found that parents had to readjust their vision of what family life would look like; for example, one mother spoke of realizing that her daughter was unable to “do the things that a normal eight‐year‐old girl would love to be doing”. Similarly, Krabbenborg et al. found that parents of children with rare conditions receiving exome sequencing results became aware of “all the things that are not possible anymore” (Krabbenborg et al., 2016). Although these studies allude to parents going through a grieving process as a result of their child's condition, none specifically make this emotional process the focus of their findings. A deeper understanding of the grieving process among parents caring for a child with a rare and undiagnosed condition is essential for improving clinical management, especially since unresolved grief, e.g., in parents of children with cancer, is known to be linked to a higher risk of long‐term mental and physical health problems (Lannen et al., 2008). Genetic counselors, in particular, play a critical role in navigating any subsequent grieving and/or emotional adaptation for parents at this turning point (Helm, 2015).

This study aims to offer insights that can enhance psychosocial support for families coping with grief related to their child's rare and undiagnosed condition by striving to understand the complex mechanisms underlying grief in the context of uncertain outcomes.

### 1.4 Study context: Whole genome sequencing—the light at the end of the tunnel?

Whole genome sequencing (WGS), which was introduced into the NHS in England following the landmark 100,000 Genomes Project (NHS England *National Genomic Test Directory*, n.d.) has demonstrated clinical value for pediatric rare diseases. It achieves a diagnostic yield of 38.6% compared to 7.8% with first‐tier genetic testing/chromosome microarray analysis (Nurchis et al., 2023), with particularly high diagnostic yields for intellectual disability. WGS may improve care and treatment decisions for some diseases as well as provide answers to patients and families (van Diemen et al., 2017), and the implementation of WGS into routine clinical care is currently under further evaluation (Bagger et al., 2024). Although the diagnostic yield is much improved, the majority of patients will still remain undiagnosed. Research with parents of children who did not receive a genetic diagnosis following genomic testing experienced disappointment and sadness, loss of hope, anxiety about the future, and frustration at the lack of access to genetic information and support groups (Gurasashvili et al., 2023; Peter et al., 2022). Even with diagnosis, parents may still grapple with ongoing uncertainty associated with their child's condition and anxiety due to limited information about rare conditions, but nonetheless value having received a diagnosis (Peter et al., 2022). Some parents report benefits like acceptance, tailored care, and reduced guilt, but others experience a loss of hope or diminished peer support (Krabbenborg et al., 2016). Regardless of receiving WGS results, coping often involves finding hope parallel to the ongoing uncertainty (Neustadt et al., 2020), underscoring the limitations of a diagnosis when the prognosis remains unclear.

## 2 METHODOLOGY

### 2.1 Study design

To obtain rich, in‐depth data, a semistructured interview style was adopted to enable participants to express their perspectives in their own words (Burgess, 1984). The exploratory study design allowed looking into further clarification of subject experience (Barroga & Matanguihan, 2022). We employed reflexive thematic analysis (Braun & Clarke, 2019) to analyze the qualitative data, an approach that recognizes the central role of the researcher's interpretation in generating themes. Accordingly, the Results section includes both descriptive summaries and interpretative commentary, supported by participants' own words as evidence, to demonstrate how our interpretations were grounded in the data. During the analysis, we identified a substantial portion of the dataset that did not align with the initial central organizing concept related to parents' identity crisis and emotional strain (separate analysis). In line with the iterative and reflexive nature of thematic analysis, we analyzed this divergent data separately. Through this process, we generated a secondary central organizing concept that offered distinct yet meaningful insights into participants' experience related to how parents cope with grief amidst the uncertainty surrounding their child's condition. This article details the findings from that analysis.

### 2.2 Data sampling

As part of the mixed‐methods study, a cohort of 394 parents of (alive) children with undiagnosed and rare conditions, recruited from across seven NHS Hospital Trusts, completed a survey within 4 weeks of providing consent for WGS (and prior to results being received; Patel et al., 2025a, 2025b). At the end of the survey, parents were asked if they would be willing to take part in a follow‐up interview. Interview participants were purposively sampled based on their scores from three validated measures assessing anxiety and quality of life, namely:

- The Generalized Anxiety Disorder‐7 (GAD‐7) Scale (Spitzer et al., 2006) which is a seven‐item measure that assesses the frequency with which the responder has experienced symptoms of anxiety over the past 2 weeks,
- The Pediatric Quality of Life Questionnaire Family Impact Module (PedsQL Family Impact Module), which assess quality‐of‐life and family impact from a physical, emotional, social, cognitive, communication, and worry perspective (Varni et al., 2004),
- The Brief Resilience Scale (BRS) (Smith et al., 2008) which relates to the responders' ability to cope or recover from stress.

Research on rare diseases highlights that parents often experience grief and loss, such as the loss of perceived normality the loss of hope, or anticipatory grief for an unrealized future, in response to their child's condition (Gurasashvili et al., 2023; Nevin et al., 2022). Our particular interest lay in the group of parents identified to be coping less well than others in similar circumstances, to understand their lived experience in more detail and to provide much‐needed evidence for clinicians and/or other health professionals/support organizations working in this area. The three measures discussed above were judged to provide a multi‐dimensional view of parents experiencing higher psychological burden; high anxiety levels reflect significant psychological distress, while lower family impact scores suggest that the child's condition substantially disrupts family life and parental functioning. Lower resilience indicates a reduced capacity to recover from or adapt to stress. Therefore, the majority of our parents had scored moderate to severe anxiety on the GAD‐7 (in the overall sample 55.1% were categorized as having minimal anxiety, 23.0% had mild anxiety, 11.4% moderate anxiety, and 10.5% severe anxiety); and/or lower scores on the PedsQL Family Impact Module indicating lower functioning (in the overall sample 8.2% were categorized as having low family functioning, 35.8% medium family functioning, and 56.1% high family functioning); and/or lower scores on the Brief Resilience Scale indicating lower resilience (in the overall sample 28.1% parents were categorized as having low resilience, 63.9% as normal resilience and 8.1% as high resilience). We also selected participants across a range of demographic characteristics (ethnicity, socioeconomic background, geographical location) as well as parents of children from a range of disease categories.

Overall, 74 potential participants who had poorer outcomes on the above‐mentioned measures were from a range of demographic backgrounds, and had indicated on their survey that they were willing to take part in an interview, were contacted (see Data S1—email invite). This resulted in 27 parents considering participation, 11 who actively declined and 36 who did not respond to two email or text invitations. The final sample consisted of 24 of 27 parents being included in the study; three declined further participation due to unforeseen circumstances.

### 2.3 Data collection

The initial idea and focus for the study (i.e., focusing on the mental health experience of parents) was discussed with an advisory team that support the overall mixed‐methods study by agreeing on study aims and objectives, reviewing and commenting on data collection tools, and supporting the interpretation of findings. The advisory team includes parents of undiagnosed children, clinicians, genetic counselors, and researchers. During that meeting, some initial suggestions for questions in the topic guide were put forward. The authors CL (a behavioral scientist and qualitative researcher with a background in rare disease and genetics) and BF (a research psychologist with a background in health communication, service evaluation and mixed methods research) developed the first draft of the topic guide for the semistructured interviews which was then discussed with the founder of Rareminds, a UK non‐profit organization specializing in rare condition psychological counseling (*About Us ‐ Rareminds*, 2020) along with a researcher from the organization, to ensure the questions were appropriate and sensitively worded. The author TMH underwent short psychological training with a psychological therapist specializing in rare disease counseling from Rareminds, to discuss addressing sensitive questions from the topic guide during interviews. Minor changes were also proposed to further improve wording.

The topic guide addressed questions around parents' health and wellbeing including the emotional impact of caring for a child with an undiagnosed and rare condition, whether and how they manage their mental health, how this has changed over time, the impact of the child's condition on relationships and what they perceive the impact of a diagnosis would be on their mental health and wellbeing (see Data S1—topic guide). Participants were offered a £10 voucher for their participation and the option of accessing psychological support from a therapist was made available in those cases where parents became visibly distressed during the interview (accessed by one parent).

In total, 24 interviews, lasting 20–100 min (mean = 55 min), were conducted (*n* = 7 were conducted by BF; *n* = 17 were conducted by TMH) and recorded using encrypted audio devices or virtual software and transcribed verbatim. Following best practice guidelines (Braun & Clarke, 2021b), interviews were conducted alongside data analysis, prioritizing data quality over achieving data saturation. Decisions about data sufficiency were guided by considerations of quality and information richness rather than the pursuit of saturation.

### 2.4 Research paradigm

Following recently published guidelines by Braun and Clarke (2025), we outline the theoretical orientation of our work in this paragraph and describe the practical application. Our research is situated within an interpretivist and post‐positivist research paradigm (Howell, 2013), viewing knowledge as contextual and constructed through interaction between researcher and participant, thus embracing researcher subjectivity as an analytic resource rather than a bias to be eliminated (Braun & Clarke, 2024a). In accordance with established guidelines (Braun & Clarke, 2021a, 2024b), author TMH, who conducted data collection (*n* = 17/24), coding and interpretation, engaged in reflexive journaling and created mind maps to document and share her thought processes. TMH reflected on her perspective as a female researcher with limited background in the clinical management of a child with a rare condition. To enhance data credibility, TMH followed recommendations for researchers new to a field (Berger, 2015), receiving regular guidance through fortnightly debriefs with her supervisor (CL), as well as incorporating valuable input from a psychologist from Rareminds (*About Us ‐ Rareminds*, 2020) and the study advisory team. While we present our analysis as one plausible interpretation of the interviews, we recognize that other scholars might draw alternative conclusions from the same data.

### 2.5 Data analysis

Thematic analysis is grounded in a reflexive and iterative engagement with the data, which allows for the generation of themes that reflect the richness and complexity of participants' experiences. Throughout this process, we maintained a flexible stance, ensuring that our analytic approach was responsive to the data as it emerged. After data familiarization, three sets of coding were applied, then grouped into patterns of meaning. During the second coding, the iterative process of creating mind maps based on the generated codes led to the creation of two central organizing concepts, one focused on identity and emotional strain (which will be the focus of a separate paper), and another on how parents cope with grief amidst the uncertainty surrounding their child's condition. At this point, these concepts were analyzed independently; however, they provide complementary insights into the phenomenon under study. To determine that the depth of exploration was sufficient, we conducted line‐by‐line coding across all transcripts, continuously compared codes and themes across participants, and discussed interpretations among multiple researchers until consensus was achieved. Analysis continued until no new codes or patterns emerged, suggesting that our analysis had sufficient information power (Malterud et al., 2016). By engaging deeply with this data, we ensured that the themes reflected the diversity and complexity of the dataset, rather than being constrained by a singular focus.

In terms of reflexivity, to support the interpretation of the preliminary results, initial findings from the reflexive thematic analysis were presented by the authors TMH and CL and discussed with the founder of Rareminds (*About Us ‐ Rareminds*, 2020) and a researcher from the same organization. Discussions highlighted in particular the ambivalence inherent in representations of societal ideals and the heroic archetype often associated with parents taking on significant caring responsibilities. In addition, the discussion also focused on the nature of grief for some parents including how it may change and adapt over time but may never leave completely. The discussion informed the choice of wording for themes and the title of the central organizing concept.

At the bi‐annual meeting of the advisory team, the results of the then later stage analysis were presented and discussed by the author TMH. Minor changes were suggested in terms of reordering the themes within the central organizing concept, with a view to what the advisory team felt was most important in terms of clinical and psychological practice and most meaningful to parents.

Qualitative analysis was facilitated through the Nvivo14 software. Descriptive statistics were generated using SPSS mac 29.0.2.0. PedQL scores were calculated according to the published protocol (Varni et al., 2004).

### 2.6 Parent involvement and advisory team

The advisory team meets biannually and agrees on study design and research aims, inputs into data collection tools, and provides feedback and interpretation of study findings. Their role is to ensure the study's alignment with the research objectives and the needs of the target population.

## 3 RESULTS

### 3.1 Participants' characteristics

The study included 24 participants (4 men, 20 women) aged 21–54 years (mean age = 38 years), most of whom identified as white British (Table 1). At the time of the interview, four parents had recently received a result from WGS; in two cases, it was diagnostic, and in two cases, it was a “no finding” result.

**TABLE 1:** Participants' characteristics.

	(*n* = 24)	%
Gender		
Female (*n*, %)	20	83.3
Male (*n*, %)	4	16.7
Age		
Age in years (mean; range)	38; 21–54	
Socioeconomical characteristics		
Income (n, %)		
Below £10,000	2	8.3
£10,001 to £30,000	6	25.0
£30,001 to £50,000	4	16.7
£50,001 to £70,000	7	29.2
Over £70,001	3	12.5
Prefer not to say	2	8.3
Ethnicity (n, %)		
White or White British	17	70.8
Asian or Asian British	5	20.8
Other ethnic group	1	4.2
Prefer not to say	1	4.2
Religion (*n*, %)		
None	10	41.7
Christian/Catholic	8	33.3
Muslim	3	12.5
Hindu	1	4.2
Sikh	1	4.2
Prefer not to say	1	4.2

Participants reported a moderate mean anxiety score (12.8, range 0–21) and low mean resilience (2.7, range 1–4). Cognitive functioning was the most impacted aspect of the PedsQL Family Impact Module, followed by family relationships and communication (Table 2).

**TABLE 2:** Participants' psychosocial characteristics.

	(*n* = 24)	%
Psychological questionnaire results		
Anxiety		
GAD‐7 score for anxiety^a^ (mean, range)	12.8; 0–21	
GAD‐7 score category for anxiety^b^ (*n*, %)		
Low	2	8.3
Mild	7	29.2
Moderate	4	16.7
Severe	11	45.8
Resilience		
Brief Resilience Scale score^c^ (mean, range)	2.7; 1–4	
Resilience scale category (*n*, %)		
Low	11	45.8
Normal	13	54.2
High	0	0
PedQl TM Family Impact Module^d^		
Total Score^e^ (mean, range)	43; 10–80	
Subscores^f^ (mean, range)		
Physical functioning (max. 30 points)	14.5; 0–23	
Emotional functioning (max. 25 points)	13.0; 0–20	
Social functioning (max. 20 points)	8.5; 0–15	
Cognitive functioning (max. 25 points)	9.4; 0–20	
Communication (max. 15 points)	6.6; 0–12	
Worry^c^ (max. 25 points)	12.6; 0–20	
Daily activities (max. 15 points)	7.5; 0–12	
Family relationships (max. 25 points)	8.9; 0–18	

^a^ Generalized Anxiety Disease Assessment based on 7 items with 0 to a maximum of 21 points.

^b^ Disease severity categories assigned to sum of points: 0–4 “low”; 5–10 “mild”; 10–14 “moderate”; 15–21 “severe.”

^c^ Brief resilience score categories: 1–2.99 “low”; 3–4.3 “medium”, 4.31–5.00 “high”.

^d^ Numbers based on *n* = 23 responses due to missing response.

^e^ Family Impact Module Score is based on 36 items encompassing 8 scales with a total score from 0 to a maximum of 100 points with higher scores indicating better family functioning.

^f^ Subscores: possible responses: 1 = “never”; 2 = “almost never”; 3 = “sometimes”; 4 = “often”; 5 = “almost always”; higher scores indicate greater impact.

Developmental conditions were the most frequently reported conditions among participants' children (Table 3). Most participants rated the child's condition as being serious (mean 3.7) and having a significant impact on their child's life (mean = 4.1; Table 3).

**TABLE 3:** Characteristics of participants' children and their disease.

	(*n* = 24)	%
Age in years (mean; range)	4; 1–15	
Disease category of child's condition (*n*, %)		
Developmental disorder	13	54.2
Developmental disorder and neurological disorder	1	4.2
Musculoskeletal	1	4.2
Neurological disorder	5	20.8
Neurological disorder and musculoskeletal	2	8.3
Ophthalmological disorder	1	4.2
Ophthalmological disorder and metabolic disorder	1	4.2
Severity of the condition^a^ (mean)		
My child's condition is serious		3.7
My child's condition has major consequences on their life		4.1

^a^ Self‐reported questions on the severity of the condition, possible answers were: “strongly disagree” = 1; “disagree” = 2; “neither agree nor disagree” = 3; “agree” = 4; “strongly agree” = 5.

### 3.2 Qualitative results

We identified three themes centred around a core organizing concept, which we named Navigating Grief Within The Context of Uncertainty, to capture the lived experiences of parents experiencing poorer emotional wellbeing who are caring for a child with a rare and undiagnosed condition.

Data from all interviews supported all themes. To illustrate these findings, the analysis section includes representative quotes from participants, with pseudonyms used in place of real names.

#### 3.2.1 Navigating grief in the context of uncertainty

The journey associated with a rare disease varies considerably across families. For some, an unexpected diagnosis brings an immediate shock, while others gradually sense that something about their child's development feels different than expected. Regardless of the pathway, both scenarios share an element of unforeseen circumstances, where parents are suddenly placed in an alternate reality that deviates from the envisioned path they had for their family. Despite their profound love for their child, many parents in our cohort grieve the loss of the imagined future, struggling to distance themselves from these anticipated visions. This grieving process is further complicated by the persistent uncertainties families face regarding their child's condition, which in turn may add to the difficulties families face when reconstructing a cohesive family narrative. Moreover, the grief lacks a clear resolution point within the context of an uncertain prognosis, and may wax and wane across the life course, thus lacking a defined endpoint. For some parents, this grief becomes backgrounded, managed, or transformed; for others, it remains more present. Over time, as parents adapt to their new reality, they find ways to navigate the unknown and establish meaning within this transformed family identity. Yet they frequently oscillate between grief and acceptance, mourning what could have been and celebrating what is. Our findings highlight the complex, dynamic nature of grief as families care for a child with a rare and undiagnosed condition.

#### 3.2.2 Theme 1: Early and recurring emotional reactions including philosophical questioning

This theme explores the challenging emotional and cognitive responses parents described as they adjust to the altered trajectory of their family life. Many expressed feelings of anger, guilt, frustration, isolation, and powerlessness, which in some cases were associated with readjusting to what family life would look like. These emotions often surfaced at the point of realization that something was different or in the early stages of searching for a diagnosis, but they were also described as recurring, particularly during difficult periods. Concurrently, some parents began philosophically questioning why this had happened to them, reflecting ongoing attempts to make sense of their circumstances.

Parents frequently described a sense of being constrained or limited in what they were able to do as a family, often in comparison to other families. Victor's reflection captures how practical decisions related to his child's care shaped where his family could live:

…our living circumstances are not ideal, we're in a flat that's too small, we're in a location that's, kind of, anything else that's bigger is unaffordable for us, but we sort of have to live here because we've found this quite good school which seems to suit my [child]'s needs, so now we're tied to [inner city], but it means that we can't do again what our peer group with neuro typical kids are doing, which is moving out to the suburbs and having lovely gardens and days in the park and everything else. We're stuck in an inner city environment… [Victor]

Words such as “tied to” and “stuck” suggest Victor feels he lacks agency in his specific circumstances. Similarly, Laura echoes feelings of isolation and restriction, again, especially when comparing herself to other families, whom she sees as being able to take part in simple weekend activities:

…And that lack of freedom really weighs on me very heavily…we're really quite isolated…we would really love to be able to do things as a family, you know, and it is very difficult, especially with social media, which I know obviously is all smoke and mirrors, but still it can feel very difficult when other families are out at the weekends doing things, even just a walk around the park … [Laura]

These accounts illustrate how grief can be intensified when parents compare their lives to the perceived flexibility and spontaneity enjoyed by other families.

Others reflected on the challenging emotions they had experienced. Susan describes how at times the emotional weight becomes overwhelming:

…There have been points when I've just wanted to walk out. And I've literally been like, I could just walk out now and not come back…. [Susan]

Susan's words illustrate how moments of exhaustion can resurface periodically rather than resolve. Other parents describe renewed grief when medical appointments do not provide the hoped‐for outcomes, such as Ingrid who describes that:

the days following when you've had contact with the hospital and maybe hadn't had the news that you were hoping… those days are particularly hard. [Ingrid]

Her account highlights the cyclical nature of grief, in which new disappointments trigger fresh waves of sadness and disorientation. Ingrid's comment “…it makes me feel angry that my life has changed massively…” can be perceived as conveying a deep sense of disruption and displacement, where life has shifted in ways she did not anticipate.

Alongside these difficult emotions, parents also engaged in a process of philosophical questioning. Laura shares with us:

…I felt like ‘why me’ and then I get frustrated because I think, ‘well why not you’? And it's really hard to sort of quantify it in your brain… [Laura]

Laura's comment reflects the tension between searching for meaning (wanting to understand “why”) and confronting the randomness of life events (recognizing that it could happen to anyone). This oscillation appears central to a process of meaning‐making, especially in contexts where no clear cause or resolution exists. Other parents also internalize these questions in their search for meaning, including wondering whether they might have done something to “cause” their child's condition. Samipa reflects:

…Yes, I do get angry, it's like every mother, every father, ‘why me’ is the question, but the thing is we don't question when we get good things in our life we don't question it, but when there's bad things we do question it, so I am angry thinking that did I do something wrong in my pregnancy… [Samipa]

Here, Samipa acknowledges her anger as being a valid emotional response to her situation. She, however, extends this thought by questioning whether she did something *“wrong”* during her pregnancy. Her statement reflects a feeling of guilt alongside the need for answers, suggesting that her anger is directed inward. Samipa tries to anchor her question “why me” in a reminder that all life is random and yet she can't help but focus on anger and self‐blame. Nilay expresses a similar sentiment:

I used to think oh maybe it's the bleeding and that was my fault, maybe I could have done something, even though I did used to go to hospital all the time. [Nilay]

She negotiates with herself, saying on the one hand that she feels guilty that she may somehow have been responsible for her child's condition, but on the other hand, she tries to reduce the intensity of her guilt by saying that she took action by going to the hospital. These reflections suggest that parents' emotional reactions were not only expressions of the challenges but also attempts to construct meaning around their experience.

#### 3.2.3 Theme 2: The parallel dream universe

This theme explores the dynamics between parents' anticipated life with their child and the reality they come to face. Several parents described how their understanding of what life would be like changed significantly when their child's condition became apparent. Victor reflects on the divergence between his imagined and actual experience as a parent:

…We dream that he speaks and then we wake up and there's the realisation that he has never said mummy or daddy or similar communications. And although we are making some progress in terms of him using a speech board to indicate what he wants, his behaviours…, [are] nothing like what we expected when we thought we'd have a healthy, happy son… [Victor]

Victor's use of “dream” could be seen as operating on two levels: both literal night‐time dreams where his son communicates, and metaphorical dreams of the child and fatherhood he once imagined. This dual meaning captures the tension and ongoing negotiation between involuntary imaginings and the lived reality of raising a child with complex communication needs. While he acknowledges his child's progress, he also shares feelings of frustration and emotional fatigue. Grief in this context could be interpreted as being shaped by the unpredictability of developmental outcomes.

He extends this reflection by alluding to the divergence between the envisioned life he thought he would have with his son and the actual experience he is having as a parent, including the impact his son's condition has had on family relationships:

…I just mourn this idea in my head of the kid I would kick a football with…I could say loads of lovely things about him as well, but it always comes down to what is quite literally in our dreams, you know, at night involuntarily or in the background during the day and then what is here when we wake up that there's just a massive difference and it affects everybody's interpersonal relationships… [Victor]

Here, Victor articulates the emotional dissonance between long‐held ideas of fatherhood and the realities of parenting a child with additional support needs. His remark that “I could say loads of lovely things about him” foregrounds the affection and pride he feels alongside grief, and the enduring impact of his son's condition on his own identity and across the family system.

Similarly, Harroop reflects on the unexpected nature of her experience. For her, the possibility of having a child with a rare or undiagnosed condition had not been part of her prior frame of reference.

I have a child that's older than my daughter and I didn't have any complications with him, he's a healthy child who hasn't had any issues like physically, mentally or anything like that. So when you have another one, or even if it's just your first, you don't really expect to go down the route of oh there's a possibility of something actually happening to them… [Harroop]

Her statement suggests how the diagnosis came as an unexpected shift from what she had anticipated. This unexpected circumstance left her unprepared for the *“complications”* she would need to navigate to support her child.

Some parents expressed particular moments of sadness or disappointment related to developmental milestones their child did not reach, particularly in contrast to their experiences with other children or societal expectations. Emily expresses feeling *“sad”*, in relation to watching her child face limitations not experienced by her peers:

…it makes me sad that she's not going to have the life that we thought she was going to have, it makes me sad when she wants to, you know, do all the things that her friends want to do and she can't do it… [Emily]

By comparing what her daughter can do with others, Emily also articulates the ongoing limitations and exclusion her daughter faces in accessing typical childhood experiences. Her sadness is intrinsically linked to this social context: it is not just about her child's limitations, but about the constant comparison to normative developmental milestones and peer activities. Her sadness is also grounded in love—she wants her daughter to enjoy the same opportunities as her peers. Similarly, Mary speaks about milestones her daughter had not reached:

…She hasn't done many ‘firsts’. She can't ride a bike without stabilisers. She doesn't get to go to birthday parties because most of them are soft play or rock up at the wall and she can't put a harness on…So yeah, that side of stuff is sad that she's missed that

Mary's reflection highlights how the divergence between her daughter's experiences and typical childhood milestones contributes to a sense of ongoing loss and reinforces the contextual nature of parental grief.

#### 3.2.4 Theme 3: Meaning‐making and moving towards acceptance

Themes 1 and 2 reflected the emotional strain and disorientation experienced by many parents. Theme 3 explores a shift wherein parents begin to reframe their thinking in a way that allows for greater emotional resilience and acceptance. Rather than resisting the unknown, they start to gradually make peace with it, and reconstruct meaning by moving towards a more constructive and hopeful mindset. This stage is not about eliminating uncertainty, but about embracing it differently.

Harroop describes a gradual shift in her thinking by saying:

…I think the longer it's gone on for the more I'm starting to understand, accept things and then just take it onboard… [Harroop]

Her comment reflects how acceptance may emerge over time, rather than from a single event or turning point. For Barbara, a spiritual encounter led to her reframing her experience and offered a way to find purpose and meaning in caring for her child:

But I went on the spiritual path, …there was a woman that had never met me before and I was struggling at the time with [child], …and she came up crying and she says ‘I just need to tell you…’, and apparently she was a spiritualist, she just went ‘I've got so many angels round me talking to me at this moment in time, I'm crying now because I'm so overwhelmed, you've got a little son that was sent to you and he was sent to you for a reason…that was kind of a turning point in my life. [Barbara]

Spirituality seems to provide Barbara with a framework that helped her interpret her caregiving experience in a meaningful way. This spiritual encounter can be seen as a turning point in her emotional journey. However, this was not always the case as highlighted by Anjali who found it “frustrating” when people would make comments such as “it's in God's hands.”

Other parents reframed their circumstances and constructed meaning by focusing on their child's strengths and resilience. For example, Mary speaks of the pride she has for her daughter:

But on the flipside she is so strong and determined and she wouldn't probably be that person had she not have had to battle and gone through what she's gone through. [Mary]

Mary's words highlight a shift in focus—from what her daughter could not do to a recognition of her determination and growth. The phrase “on the flipside” signals a shift in Mary's perspective and reflects an active effort to reframe a difficult experience through the reconstruction of meaning. This reframing can be seen as enabling Mary to integrate her daughter's challenges into a more coherent and positive narrative, whereby challenges and resilience coexist.

Similarly, Laura describes how her emotional response has evolved over time:

…So I don't grieve as much as I used to for what I would have hoped for him to accomplish for his own good, not from any sense of vanity on my part, and that's I think partly because he did start school in September, he loves school, he has really learned so many new skills, he comes home happy and fulfilled… [Laura]

Laura's shift in focus towards her son's progress and happiness seems to reduce what could be described as her earlier sense of loss. Her reflection emphasizes that adaptation can be shaped by the child's own experiences and milestones. The fact that “he comes home happy and fulfilled” highlights that her child's happiness is her main priority, and this provides a sense of peace and emotional fulfillment. Anjali expresses a similar sentiment:

…so he himself has changed our way of thinking because he's just always so happy and he loves us both to pieces and what more could you ask for … he's obviously not unhappy, so I think you think if he's being like that then we should be happy, let's enjoy our lives together [Anjali]

Again, here we see a shift from sorrow to acceptance, prompted by the joy and love expressed by the child. Rather than being anchored in “anxiety” and “frustration” around the child's condition, Anjali's perspective moves toward what is present: love, connection, and her son's capacity for joy.

Many parents were not able to articulate a specific incident that helped them, but described acceptance as something that emerged with time:

…I think it just is what it is isn't it? You just learn to live with it… [Louise]

For Louise and others, adaptation did not come from active reframing, but from learning to integrate new realities into daily life.

Nilay describes how whilst sometimes she finds her circumstances overwhelming, she finds it within her to cope:

Sometimes it gets a bit too much and you're like oh my god I can't do it and then you're like no I have to be strong to be able to do it, so that's just hard. [Nilay]

Nilay's reflection illustrates the cyclical nature of grief, showing how parents oscillate between moments of emotional overwhelm (“I can't do it”) and the necessity of resilience (“I have to be strong”), highlighting that adaptation is a dynamic, context‐dependent process rather than a linear progression. She goes on to say:

“a lot of times you just have to have patience to be honest, and then also thank God every day for the support I have… everyone keeps really positive… on a day when I feel really down I just call my mum or my partner and they just tell me, like, OK it's fine” [Nilay]

Nilay's comment highlights how she has learned to adapt to her circumstances, and in particular, acknowledges the importance of patience and emotional support. Her comment also illustrates how emotional regulation was often supported by close family members and faith, which helped her manage moments of distress.

Several parents described the role of psychological and peer support in helping them cope. For example, Anna shared:

I've had lots of different cognitive behavioural therapy … I feel a lot better now, so that's good. [Anna]

Anna's reflection speaks to the positive impact that cognitive behavioral therapy has had on her mental health. At the same time, her phrasing (“lots of different…”) implicitly points to the prolonged and potentially challenging nature of the therapeutic process, suggesting that improvement was neither immediate nor straightforward.

Other sources of support mentioned by participants included peer support groups, where parents can meet other parents of children with rare and undiagnosed conditions. These appeared to play a pivotal role in helping them cope. Emma spoke about the importance of connecting with other parents going through a similar experience:

They can empathise with you and they're walking in the same shoes as you…. [Emma]

“Walking in the same shoes as you” suggests there is comfort for Emma in having others validate the feelings she is experiencing.

Peer support groups were often seen as valuable spaces where parents could speak openly and feel understood. However, some parents noted difficulties in accessing peer support groups, mentioning a lack of information about how to engage with these services or the absence of appropriate groups due to the rarity of their child's condition.

## 4 DISCUSSION

### 4.1 Summary

We selectively sampled parents who were experiencing moderate to severe anxiety and/or low quality of life/family functioning to understand how this particular cohort of individuals was coping with the experience of caring for a child with a rare and undiagnosed condition. Our findings reveal that theirs is a complex and emotional journey, characterized by grief, acceptance, and adaptation. The shock of shifting from an envisioned life trajectory to becoming a caregiver, combined with the lack of diagnosis and prognosis, can bring with it feelings of entrapment, frustration, guilt, and loss. There are often competing emotions at play, involving the dual reality of love for their child alongside the challenges of raising a child with complex needs, particularly against a background of broader systemic failures to adequately support families and recognize diverse developmental trajectories. The absence of closure, due to the undiagnosed nature of the conditions affecting children in our study, added to the cyclical nature of grief which occurred due to context‐dependent triggers, for example, intense emotional periods or missed developmental milestones. However, over time, many parents find a balance between grief and acceptance. Support systems, spirituality, gratitude, patience, and the ability to reframe challenges all play key roles in helping parents cultivate meaning and resilience.

Many of the emotional as well as practical challenges described by parents in this study can be viewed as manifestations of substantial unmet support needs within a wider systems context. Consistent with Wilsnack et al. (2024), many of the experiences voiced by parents—including uncertainty, emotional exhaustion, isolation, and grief—reflect not only their personal lived experience of caregiving but also the failure of the health and social care systems to provide timely, coordinated, and comprehensive support. Parents frequently described a lack of access to specialist care, inadequate recognition of their mental health needs, insufficient financial or respite support, and limited peer connection. These gaps left families feeling overwhelmed and alone in navigating complex and demanding caregiving roles. Rather than viewing parental grief as an internalized response to their circumstances, our findings support a broader interpretation: that such grief and distress often emerge from, and are exacerbated by, systemic neglect and marginalization of families caring for children with rare and undiagnosed conditions. Our findings echo those from a recent report from Genetic Alliance UK which highlighted how barriers or delays accessing care for people with rare conditions can impact other areas of life including loss of earnings, disruption to school and work, as well as an emotional burden that can affect mental health (The Policy Team at Genetic Alliance UK, 2023).

As Breen and O'Connor emphasize (Breen & O'Connor, 2007), grief should not be oversimplified into categories of “normal” versus “complicated.” Instead, it must be situated within the specific historical, social, and medical contexts in which it arises. In our study, the salient contextual factors included prognostic ambiguity, the absence of a definitive diagnosis, and the ongoing challenges of parenting within a healthcare system often unprepared to meet their needs. This context shaped the parents' grief as dynamic, oscillating, and continually renegotiated across the life course. By framing our findings in this way, we align with recommendations to move beyond rigid grief typologies and to foreground the unique circumstances that shape families' experiences.

Some of the emotional experiences described by the parents in this study—uncertainty, guilt, isolation, hopelessness, and shock—resemble the experiences of parents whose child has a life‐threatening condition, such as mothers caring for a child with cancer or other rare but diagnosed conditions (e.g., muscular dystrophy, heart defects; Najafi et al., 2022). The parents in our study experience grief within a different context, which may also apply when the prognosis and even the diagnosis are unclear. Unlike children with a life‐threatening prognosis, many rare and undiagnosed conditions are not life‐threatening, and as we found in this study, parents learn to live with the uncertainty around their child's prognosis over time. In fact, research with a broader sample of parents of children with undiagnosed conditions has found that the importance placed on finding a diagnosis wanes as their child gets older, and that ultimately parents tend to accept the situation and ‘get on with life’ (Skirton et al., 2010).

Our findings align with a number of well‐established theories around grief. Ambiguous Loss Theory (Boss, 1999) states that ambiguous loss occurs when there is an unclear or unresolved loss leading to feelings of grief without closure. Our parents could be described as having experienced ambiguous loss in that there was the loss of the imagined future, but also the absence of a diagnosis, creating uncertainty about what the future holds. This aligns with findings from other literature which describe ambiguous loss as a key aspect of parental grief in the context of some rare and undiagnosed conditions (Kurtzer‐White & Luterman, 2003; O'Brien, 2007). In addition, our findings also align with the Dual‐Process Model of Coping With Bereavement (Stroebe & Schut, 2010) which posits that grief involves two concurrent processes: loss‐orientated processes, such as with parents in our study mourning the loss of a child who they could “kick a football around,” and restoration‐orientated processes, which focus on adapting to new realities or redefining expectations of their child's future. Our findings also resonate with the meaning‐centred model of grief (Gillies & Neimeyer, 2006) which emphasizes grief as an ongoing process of meaning reconstruction rather than a state to be resolved. Parents in our study described how their grief often resurfaced when faced with new challenges, such as yet unmet developmental milestones or medical appointments that did not yield the hoped‐for results, while also finding ways to reframe their experiences through acceptance and meaning‐making. This reflects what the model describes as a continual phase of grief, in which bereaved individuals (or, in this case, parents experiencing grief without a defined endpoint) repeatedly negotiate meaning as circumstances shift. By situating grief within the broader context of uncertainty surrounding rare and undiagnosed conditions, our study highlights how parents' emotional journeys involve both cyclical returns of grief and evolving pathways of adaptation.

The unique contribution of our study is in bridging these various theories of grief by showing how they explain different aspects of the experiences of parents of children with rare and undiagnosed conditions who have poorer emotional wellbeing (moderate/high anxiety or reduced quality of life) compared to other parents. Unlike bereavement contexts, where grief often (but not always) follows a more defined trajectory, parents in our study faced a grief process complicated by diagnostic uncertainty, ambiguous prognosis, and the ongoing demands of caregiving. Their accounts illustrate how grief, hope, and resilience often coexist as interwoven processes, rather than discrete or opposing states. In doing so, our study expands existing grief frameworks by showing how meaning‐making and adaptation unfold for families navigating rare and undiagnosed conditions.

Recent psychological theories, including revisions to the hedonic treadmill model, suggest that individuals do not always return to a fixed baseline of well‐being after major life events (Diener et al., 2006). Instead, set points for emotional well‐being can vary, shift over time, and differ across emotional and cognitive dimensions of well‐being. This may help explain parents' descriptions of gradually “learning to live with it” or adapting over time. Positive adaptation after perceived loss or (emotional) trauma may follow multiple, individualized pathways (Bonanno, 2004), shifting the focus to support strategies.

Our findings can support the development of recommendations for practices for supporting parents who may be struggling to process their new life path (see below). Finally, and perhaps most importantly, our study gives a voice to parents of children with rare and undiagnosed conditions who are experiencing poorer emotional health, a group which has up until now been overlooked in the grief and caregiving literature. Our study contributes to a more inclusive understanding of the ongoing, complex, and diverse ways that grief is experienced by parents of children with rare and undiagnosed conditions.

### 4.2 Recommendations for practice

Based on our findings, we recommend the following aspects to healthcare professionals involved in the clinical management of children with a rare and undiagnosed condition:

1. *Recognize chronic sorrow and ambiguous loss*: Parents may not communicate freely about their expectations and disappointments related to their imagined parenthood. O'Brien et al. propose that, regardless of a child's diagnosis, symptoms of grief tend to persist longer and manifest more intensely when mothers experience greater uncertainty about their own identities (O'Brien, 2007). It is important, therefore, that health professionals understand the emotional challenges that these parents face, including the concepts of chronic sorrow and ambiguous loss, so that they are better equipped to provide compassionate and empathic care.
2. *Validate and normalize grief in the context of uncertainty*: Parents may feel isolated in their grief. Healthcare professionals should be trained to understand and acknowledge the concept of grief in the context of uncertainty and without a defined endpoint. Validating parents' recurring experiences of grief as natural and expected responses may reduce feelings of isolation. A similar recommendation was made in a study looking into mothers' grief caring for a child with predominantly neurological disabilities (Lee et al., 2022).
3. *Provide tailored and ongoing emotional support to parents*: Access to psychological support for parents coping with grief and ambiguous loss alongside other possible mixed emotions they may experience—such as love, affection, pride, joy gratitude entrapment, frustration, anger, and guilt—would be valuable. This may also help parents develop resilience and move more quickly to adaptation and acceptance.
4. *Signpost to peer support*: Support from outside the healthcare system was found to be important for some of the participants in this study. Peer support communities, such as those facilitated by organizations like Unique (Andersen et al., 2025), play a vital role in bridging the gap between diagnosis and follow‐up care by connecting families, clinicians, and researchers, and offering shared knowledge, emotional support, and lived‐experience to guide and empower those affected by rare genetic conditions. Shared experiences can reduce isolation and foster a sense of belonging. Even without a diagnosis, there are support groups, such as SWAN UK (Syndromes Without A Name), where parents can meet parents of other children with rare and undiagnosed conditions. Health professionals should signpost parents to such organizations.

### 4.3 Reflections of the first author from a reflexive standpoint

As a trainee pediatrician, I have found that the parents of children with rare conditions that I see experience many of the emotions identified through this study. After these clinical encounters, I often wonder: Have I truly communicated effectively with the parents? Do they feel less alone in their experience? What will they take away from our discussion, and how will they feel about it in the days to come? Through this research, I have been able to understand and appreciate more fully the nature of grief among these parents. It is challenging to address the sensitive topic of grief and to validate it as a normal and expected response to their situation. But as clinicians dealing with this parent population, I feel it is our duty to improve the way we support these families. I hope that this research moves us forward in that respect.

### 4.4 Strengths and limitations

We purposively sampled parents with medium to high anxiety as well as parents who had lower health‐related quality of life and family functioning, and/or lower resilience. It is therefore highly likely that these participants were experiencing grief more acutely compared with those who were less anxious and/or had higher quality of life, family functioning, and/or higher resilience. Our findings therefore need to be interpreted as reflecting a very particular group of parents at a particular timepoint (undergoing WGS). A limitation of our study was that it was quite homogenous in that most participants were white British, highlighting the need for further research involving other ethnic and population groups who may experience different or additional emotional challenges, and to specifically inform family‐integrative interventions that respond to the needs of diverse families (Broden et al., 2024). Furthermore, many participating parents had children with rare developmental and neurological disorders and therefore the findings are not necessarily representative of parents of children affected by other condition types. In addition, as participation in the study was voluntary, there remains the possibility of self‐selection bias among the parents we contacted. Those who chose to take part may differ in certain ways from those who declined, although we cannot determine the specific reasons for these differences. Consequently, the perspectives presented here may not capture the full range of experiences within the broader parent population.

Moreover, less than a quarter of our participants were fathers. Their experience of grief may be different as previous studies from pediatric cancer suggest (Pedraza et al., 2023). Further research with fathers would therefore be valuable. Finally, this study focused solely on parent perspectives and did not include the perspectives of patients themselves. This research was not intended to speak for the experiences of children, and we recognize the dynamic relationship that can exist between child and parent perspectives.

## 5 CONCLUSION

Our study highlights the complex and multifaceted nature of grief for parents of children with a rare and undiagnosed condition who experience poorer emotional wellbeing. This grief is an evolving process that reflects both the disruption of imagined futures and the challenge of navigating unsupportive social systems. By highlighting these experiences, our study also points to the need for system‐level changes to alleviate the potential challenges that amplify this grief. Our research provides a nuanced understanding of the complex phenomenon of grief and coping in this rarely heard group. Future research should continue to explore the lived experiences of parents who are experiencing poor emotional wellbeing to understand protective factors at both the micro (individual) and macro (social and cultural) levels.

## AUTHOR CONTRIBUTIONS

**Tara Maria Hoffmann:** Data curation; formal analysis; investigation; visualization; writing – original draft. **Bettina Friedrich:** Conceptualization; investigation; writing – review and editing. **Celine Lewis:** Conceptualization; funding acquisition; supervision; writing – review and editing.

## FUNDING INFORMATION

This research is funded through an National Institute for Health and Care Research Advanced Fellowship Grant (NIHR300099), awarded to Celine Lewis. The views expressed are those of the author(s) and not necessarily those of the National Institute for Health and Care Research or the Department of Health and Social Care.

## CONFLICT OF INTEREST STATEMENT

Celine Lewis, Bettina Friedrich, and Tara Maria Hoffmann have no competing interests to disclose.

## ETHICS STATEMENT

This study was reviewed and gained approval by the NHS Research Ethics Committee [London–Bloomsbury Research Ethics Committee, REC reference: 21/PR/0678]. An amendment was submitted to include my name and CV on the research ethics. Parents who wished to participate received a participant information sheet about the study and gave consent to take part in the study including for the interview to be audio‐recorded and transcribed. The option of speaking to a trained psychotherapist from Rareminds was provided to any participant who was adversely affected from taking part in the interview, with referral organized by the interviewer. It was communicated to the interviewee that they could stop or withdraw from the study at any time. The debriefing process for the interviewing researcher included regular fortnightly meetings with the PI, CL, to discuss the interview experience (de‐briefing discussions).

## CONSENT FOR PUBLICATION

Parents agreed that data can be published after removing identifying features and anonymisation. All authors agreed with this manuscript to be published.

## Supporting information


Data S1.

## Data Availability

The qualitative data from this study, consisting of anonymized interview transcripts, is not publicly available due to the sensitive nature of the topics discussed and participant consent agreements. However, excerpts of transcripts relevant to the study findings can be shared upon reasonable request to the corresponding author.
