# Supplementary material for: “I don't grieve as much as I used to”: A qualitative study on parents of children with rare and undiagnosed conditions navigating grief in the context of uncertainty
Source: J Genet Couns. 2025 Nov 29;34(6):e70149. doi: 10.1002/jgc4.70149 (PMC12664339; doi:10.1002/jgc4.70149)
Supplement: Supplementary file 1 — Data S1. [file JGC4-34-0-s001.zip › Supplementary material - email invite copy 2.docx]

Dear [name]

Many thanks for participating in our **Parent Experience Study** and for returning your survey. We appreciate you participating in our study!

As noted on the participant information sheet, we are conducting follow up interviews with some of the participants who have returned surveys. We are particularly interested in hearing about parents health and emotional wellbeing.

We are writing to you to ask whether you would be interested in doing an interview with us?

The interview takes around 40-60 min and as a token of our appreciation for your participation we would send you another Amazon voucher of £10.

Please let us know if you have any questions.

Best wishes

Parents Experience Study team
