# Supplementary material for: “I don't grieve as much as I used to”: A qualitative study on parents of children with rare and undiagnosed conditions navigating grief in the context of uncertainty
Source: J Genet Couns. 2025 Nov 29;34(6):e70149. doi: 10.1002/jgc4.70149 (PMC12664339; doi:10.1002/jgc4.70149)
Supplement: Supplementary file 1 — Data S1. [file JGC4-34-0-s001.zip › Supplementary material - topic guide copy 2.docx]

FOLLOW UP INTERVIEWS STUDY 3 – T1

Topic Guide

1. We know that caring for a child with a rare, undiagnosed condition can impact parents’ mental health and emotional wellbeing. How would you say you are currently doing in terms of your health and wellbeing?
2. How would you describe the impact your child’s condition has had on your health and wellbeing?
3. Are there any particular things you do to manage your mental health and wellbeing?
4. What if anything has been particularly helpful to manage your mental health and wellbeing?
5. Are there things you have done which you would describe as less constructive in order to manage?
6. Do you feel the way you manage your mental health has changed over the years? If yes, in what way?
7. Are you receiving any emotional support? If yes, from who?
8. Have the health professionals involved in your child’s care provided you with any emotional support?
   1. If yes, how do you feel about it?
   2. What is working?
   3. What could be done better?
9. Is there anything that can be done by the Genomic Medicine Service that would be helpful?
10. Do you feel your child's condition has an impact on family relationships? In what way? With whom in particular - partner, parents, other children?
11. Have you contacted or joined any support groups? If yes, what difference/impact if any has this made in terms of your mental health and wellbeing?
12. What difference, if any, do you think a diagnosis would have your mental health and emotional wellbeing?
13. Is there anything else that you would like to add on this subject?
